# Randomized Controlled Trial for Promotion of Healthy Eating in Older Adults by Increasing Consumption of Plant-Based Foods: Effect on Inflammatory Biomarkers

**DOI:** 10.3390/nu13113753

**Published:** 2021-10-24

**Authors:** Andreas Nilsson, Antonio Cano, Oscar Bergens, Fawzi Kadi

**Affiliations:** 1 School of Health Sciences, Örebro University, 701 82 Örebro, Sweden; oscar.bergens@oru.se (O.B.); fawzi.kadi@oru.se (F.K.); 2 Service of Obstetrics and Gynecology, Hospital Clínico Universitario-INCLIVA, 46010 Valencia, Spain; antonio.cano@uv.es; 3 Department of Pediatrics, Obstetrics and Gynecology, University of Valencia, 46010 Valencia, Spain

**Keywords:** fruit and vegetables, inflammation, diet, nutrition, physical activity, aging, TRANCE, TRAIL, CX3CL1

## Abstract

To what extent the intake of fruit and vegetables (FV) influences inflammatory status remains elusive, particularly in older populations. The aim of the present study was to determine the effect of increased FV intake for 16 weeks on circulating biomarkers of inflammation in a population of older men and women. Sixty-six participants (65–70 years) randomly assigned to either FV or control (CON) groups were instructed to increase FV intake to five servings per day through nutritional counseling (FV) or to maintain habitual diet (CON). Dietary intake and physical activity level (PA) were determined using food frequency questionnaire and accelerometers, respectively, at the start and end of the intervention. C-reactive protein (CRP), interleukin 6 (IL-6), IL-18, macrophage inflammatory protein-1α (MIP-1α), MIP-1β, tumor necrosis factor-α (TNF-α), TNF-related apoptosis-inducing ligand (TRAIL), TNF-related activation-induced cytokine (TRANCE), and C-X3-C motif chemokine ligand-1 (CX3CL1, or fractalkine) were analyzed. The FV group significantly increased daily FV intake (from 2.2 ± 1.3 to 4.2 ± 1.8 servings/day), with no change in CON. Waist circumference and PA level were unchanged by the intervention. Interaction effects (time × group, *p* < 0.05) for TRAIL, TRANCE, and CX3CL1 denoting a significant decrease (*p* < 0.05) in FV but not in CON were observed. No corresponding effects on CRP, IL6, TNF-α, MIP-1α, and β and IL-18 were observed. The present study demonstrates the influence of increased FV consumption on levels of some inflammatory biomarkers in a population of older adults. Future work is warranted to examine the clinical implications of FV-induced alterations in these inflammatory biomarkers.

## 1. Introduction

Aging is a multifactorial process characterized by a progressive decline in several physiological functions, leading to increased risk of cardiometabolic abnormalities and manifesting disease [1,2]. It is currently suggested that elevated levels of circulating inflammatory biomarkers in older adults, a phenomenon named “inflammaging”, with the presence of metabolic risk factors including central obesity, dyslipidemia, hyperglycemia and hypertension are at the very center of age-related development and progression of chronic diseases [3,4,5]

There is also mounting evidence in favor of the strong contribution of dietary habits to the development of chronic diseases. An evaluation of the impact of diet on health among adults aged 25 years and older across 195 countries revealed that overall, one fifth of deaths globally in 2017 were attributable to poor dietary habits [6]. The same report revealed that diets characterized by low fruit and vegetable (FV) consumption accounted for more than half of all diet-related deaths globally [6]. Unfortunately, several reports have highlighted low FV intakes in older adults [7,8]. Therefore, to mitigate the development of age-related chronic diseases, the promotion of healthy dietary habits including increased FV intake to five servings per day (approx. 400 g) has been endorsed by major health organizations [9].

FV are important sources of fiber and micronutrients and contains phytochemicals with anti-inflammatory properties [10]. In this respect, there is evidence for favorable relationships between FV intake and health outcomes. For example, meta-analyses of prospective cohort studies have indicated that the risk of coronary heart disease and stroke decrease by 4% for each additional portion per day of FV [11,12]. However, biological links between FV and inflammatory status remain elusive and experimental evidence about the modulation of immune function in response to increased FV intake, particularly in older populations, are inconclusive. In a population of older adults aged between 65 and 85 years, improved immunological response was observed in participants assigned to consume five servings FV/day compared to those consuming two servings FV/day [13]. Furthermore, a reduction in C-reactive protein (CRP) level, a clinically established marker of inflammation occurred in response to increased FV intakes up to eight servings per day compared to consuming two FV servings per day in a population of young adults [14]. In contrast, no significant changes in CRP level occurred in response to FV intakes up to six servings per day compared to one serving per day in a sample of middle-aged adults [15]. Interestingly, while CRP level was unaltered in response to increased FV intake to five servings per day compared to two servings per day for 16 weeks, a significant reduction in serum amyloid A, another inflammatory biomarker was observed in a sample of older adults [16]. This indicates that the exploration of the impact of FV on inflammatory status needs to consider not only clinically established markers, but also a wider set of inflammatory biomarkers accounting for the complexity of the immune function. Moreover, it is acknowledged that physical activity behavior may also alter the circulating levels of inflammatory biomarkers [17]. Therefore, assessment of physical activity level becomes crucial to determine the true effects of FV on inflammatory status.

The aim of the present study was to determine the effect of increased FV intake for 16 weeks on circulating biomarkers of inflammation in a population of older men and women.

## 2. Materials and Methods

### 2.1. Study Design

The present study is a 16-week randomized, controlled parallel-group trial. Eligible participants were randomly assigned to either fruit and vegetable (FV) or control (CON) groups. Block-randomization based on biological sex was performed to ascertain equal sex distribution in the two groups. Biological variables, PA level, and dietary intake were assessed before and after the intervention. Written informed consent was obtained from participants and all experimental procedures were performed according to the standards of the Declaration of Helsinki. The study was approved by the Swedish Ethical Review Authority (2017/511). This trial is registered at ClinicalTrials.gov (NCT 04062682).

### 2.2. Participants

Two-hundred and fifty-two community-dwelling older men and women (age range 65 to 70 years) were enrolled in the screening phase of the project through local advertisement. Inclusion criteria for the randomized controlled trial presented here were: waist circumference ≥80 cm for women and ≥94 cm for men; FV intake <5 servings/day; no use of prescribed anti-inflammatory medication. Exclusion criteria were: Body mass index (BMI) ≥35, overt disease including diabetes mellitus, coronary heart disease, musculoskeletal disorders, psychiatric disease, mobility disabilities, and food allergies.

### 2.3. Intervention

Participants in FV were instructed to increase FV intake to five servings per day, according to current guidelines for healthy eating [9]. Two group-based counseling sessions led by a nutritionist were conducted at the beginning of the 16-week intervention to support the adoption of new dietary habits. Increased FV consumption was reinforced using a behavioral support package (reading materials on guidelines, recipes, cooking tips on healthy food choices) provided through a digital platform. After two and 10 weeks during the intervention period, increased FV intake was monitored to further reinforce compliance. Participants in CON were instructed to maintain their habitual dietary intake. Importantly, both groups were instructed to maintain their habitual physical activity level.

### 2.4. Assessment of Dietary Patterns

Dietary assessments were performed before and at the end of the intervention. In order to assess habitual dietary intake, a 90-item validated food-frequency questionnaire (FFQ) was used as previously described [18]. In brief, the FFQ consists of nine fixed alternatives for the determination of intake frequency (never, occasionally, 1–3 times/month, 1 time/week, 2–3 times/week, 4–6 times/week, 1 time/day, 2–3 times/day, ≥4 times/day). In addition, we further assessed the number of FV portions per day based on the following questions: how often do you eat fruit and berries including all types of fruit and berries (fresh, frozen, preserved, juices, compote, etc.)? How often do you eat vegetables and root vegetables including all types of vegetables, legumes, and root vegetables except potatoes (fresh, frozen, preserved, stewed, vegetable juices, vegetable soups, etc.)? The following intake frequencies were used: less than one serving per day, one serving per day, two servings per day, three servings per day, four servings per day, five servings per day or more. Total energy intake, macronutrient intake (carbohydrates, fat, and protein), and FV intake were derived. Compliance to FV was evaluated before and at the end of the study period.

### 2.5. Assessment of Physical Activity

Physical activity level was determined objectively using the Actigraph GT3x monitor (Actigraph, Pensacola, Florida) at the start and by the end of the intervention period. Accelerometers were worn with an elastic belt around the hip for a whole week as previously described [19]. The monitor had to be worn for at least four days with at least 10 h per day to be included in the data analysis. Total accelerometer counts over registered time (counts per minute, CPM) were calculated and expressed as habitual physical activity level.

### 2.6. Assessment of Anthropometrical Variables

Body height and weight were measured barefoot and with light underwear using a stadiometer and a balance, respectively. BMI was determined as weight divided by squared height (kg/m^2^). Waist circumference was determined at the midpoint between the lower costal margin and the iliac crest using a measuring tape by trained personnel.

### 2.7. Assessment of Serum Levels of Inflammatory Biomarkers

Blood samples were collected in the morning between 8 AM and 9.30 AM after an overnight fast by a licensed nurse. Participants were instructed to avoid smoking and alcohol and not to engage in any strenuous physical activity 24 h before the blood sample. Blood was centrifuged at 4000 rpm for 10 min and stored at −80 °C for further analysis. The clinically established marker of inflammation, CRP, was assessed using a high-sensitivity C-reactive protein (Hs-CRP) Kit by a fully automated immunoturbidimetric assay (Advia 1800, Chemistry System, Siemens, Germany). In addition, the following inflammatory biomarkers previously implicated in progression of cardiometabolic abnormalities [20,21,22,23] were assessed using the Olink Proseek Multiplex technology (Olink, Uppsala, Sweden): interleukin-6 (IL-6), IL-18, macrophage inflammatory protein-1a (MIP-1α), MIP-1β, tumor necrosis factor-α (TNF-α), TNF-related apoptosis-inducing ligand (TRAIL), TNF-related activation-induced cytokine (TRANCE), and C-X3-C motif chemokine ligand-1 (CX3CL1, or fractalkine). Briefly, a pair of oligonucleotide-labeled antibodies are pairwise bound to the target protein present in the sample and a new PCR target sequence is formed by a proximity dependent DNA polymerization event. The resulting sequence is subsequently detected and quantified using standard RT-PCR.

### 2.8. Statistical Analysis

Data are expressed as means ± standard deviation unless otherwise indicated. Normal distribution was assessed visually and with the Shapiro–Wilk test. A two-way (within-subject: time; between-subject: group) repeated measures ANOVA (analysis of variance) was used to assess potential interaction effects (time × group) on dependent variables. When significant interactions were evident, post-hoc pairwise (pre-post) comparisons within each group were conducted with correction for multiple testing based on the Benjamini–Hochberg procedure. Change in FV intake was analyzed using the Wilcoxon paired ranks test. Based on clinical outcomes for cardiometabolic health (here CRP), power calculation indicated that moderate effect sizes can be detected with 30 participants per intervention arm, with a statistical power ≥0.8 and an alpha-level set to *p* < 0.05. Significance level was set to *p* < 0.05. All analyses were performed using SPSS version 26.

## 3. Results

**Figure 1:**
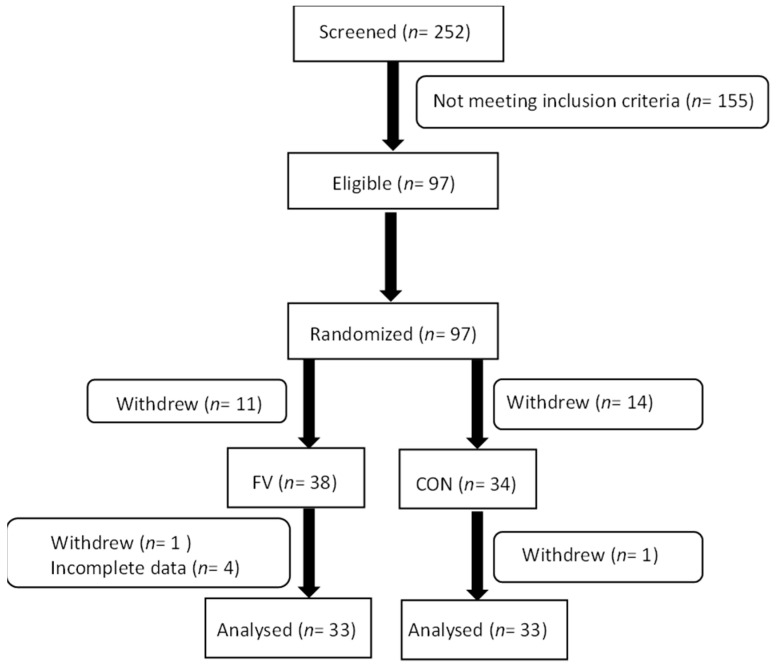
Trial flowchart showing enrolment, allocation, and analysis of participants in the RCT. FV: fruit and vegetable, CON: control.

A total of 66 subjects (FV: 67 ± 1 years; CON: 67 ±1 years) with complete data on all variables participated in all parts of the intervention (Figure 1). Forty-seven percent of participants used prescribed medication and 6% were smokers. Assessment of habitual physical activity level at baseline showed that participants accumulated an average of 328 ± 103 CPM. Anthropometry data of participants assigned to the two intervention groups are presented in Table 1.

Average number of FV servings was assessed before the start of the intervention and indicated a FV intake of 2.2 ± 1.3 servings per day for all participants. Table 2 shows FV intakes before and during the intervention period in both FV and CON groups. There were no significant group differences in FV intake before the start of the intervention.

Importantly, in response to the intervention, participants belonging to the FV group showed a significant increased number of FV servings per day, approximating twice the baseline average amount. In contrast, no corresponding change was observed in CON (Table 2). Furthermore, the changed FV intake was accompanied by significant increases in reported total energy (+10%), carbohydrate (+19%), and fiber (+26%) intakes in FV but not in CON (Table 3). The intervention-induced increased FV intake was not accompanied by significant changes in protein and fat intakes (Table 3).

In both groups, the two common body composition markers, BMI and waist circumference, did not change in response to the 16 week intervention period. Moreover, there was no significant change in average PA level between pre- and post-measurements in both FV (337 ± 105 vs. 314 ± 107 CPM) and CON (320 ± 101 vs. 308 ± 104 CPM) groups.

**Figure 2:**
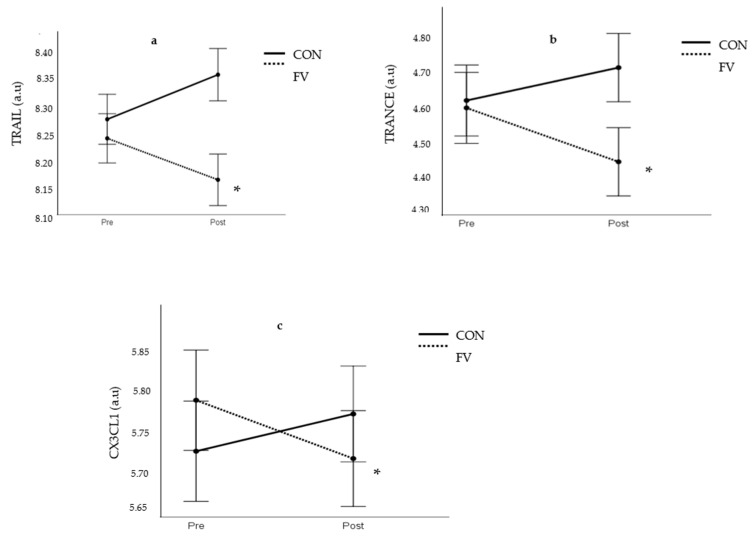
Effect of intervention on TRAIL (**a**), TRANCE (**b**), and CX3CL1 (Fractalkine) (**c**). FV; fruit and vegetable group, CON; control group. a.u; arbitrary units. * significant difference in FV (*p* < 0.05).

The analysis of inflammatory status revealed that the clinically established biomarker of inflammation CRP was not significantly changed in response to the intervention period (FV geometric mean: 1.51 ± 1.49 vs. 1.59 ± 1.18 mg/L; CON geometric mean: 1.36 ± 2.56 vs. 1.19 ± 2.10 mg/L). Interestingly, there were significant interaction effects (time × group, *p* < 0.05) with respect to the pro-inflammatory biomarkers TRAIL, TRANCE, and CX3CL1. Significant decreases (*p* < 0.05) in circulating levels of TRAIL, TRANCE, and CX3CL1 occurred in FV but not in CON (Figure 2). However, there were no corresponding interaction effects on circulating levels of the inflammatory biomarkers IL6, TNF-α, MIP-1α and β, and IL-18 (Supplementary Figure S1).

## 4. Discussion

The present randomized controlled study explored the impact of increasing FV intake on selected biomarkers of systemic inflammation in older men and women. Here, we demonstrate that participants who received dietary counseling increased the average number of daily FV servings by two-fold during the 16-week intervention. Importantly, significant reductions in circulating levels in a number of inflammatory biomarkers occurred in response to increased FV intake.

There are very few studies aiming to increase fruit and vegetable intake in older adults and determine the implications of this dietary change on the inflammatory environment. The promotion of increased FV intake in the present intervention was based on a health education approach, where a few counseling sessions led by a nutritionist were combined with support by digital tools designed to be feasible for implementation into wider health care settings. In contrast to several previous studies investigating the impact of increased FV on health outcomes [24,25,26], free-of-charge amounts of fruits and vegetables were not provided to our participants. Interestingly, a 10-week intervention based on educational counselling sessions with and without the provision of fruits and vegetables revealed a limited impact of fruit provision compared to counseling alone in increasing FV servings toward recommended amounts [27]. Furthermore, FV consumption has been shown to return to baseline levels when provision of FV ends [25,26], indicating this approach to be less optimal in terms of promoting sustainable changes in dietary habits. Our data indicate a two-fold increase in FV servings per day in participants belonging to the FV group, with no corresponding changes in the control group. This lends further support to findings from a systematic review concluding that nutritional education efforts are effective in terms of promoting healthier dietary habits [28]. Furthermore, a compilation of studies based on health counseling reported differences between intervention groups (FV group vs. control group) from 0.2 up to 2.2 servings/day [29]. Therefore, the magnitude of change in FV servings achieved by our participants in relation to the controls corresponds to the upper range of FV changes reported in studies based on similar educational approaches [29].

The present study showed that the increase in fruit and vegetable intake in the FV group was accompanied by an increased total amount of carbohydrates, with a substantial increase in fiber intake. This is in line with data from a compilation of studies showing that increased FV consumption leads to elevations in carbohydrate and fiber intakes and possibly a reduction in fat intake [30]. Notably, the between-group difference in fiber intake reached 3 g/day in favor of the FV group, which is in agreement with data previously reported on the impact of increased FV consumption on dietary nutrient intakes [29]. Additionally, a pooled data analysis showed that one portion increase in FV was associated with a modest increase in total energy intake [31], which was also further evidenced in our data of older men and women. However, this small increase in total energy intake was not accompanied by changes in either BMI or waist circumference, which is similar to what has been commonly reported in studies targeting increased FV intakes without further instructions to reduce overall energy intake [32].

The present study explored the impact of increased number of daily servings of FV on selected biomarkers of inflammation including clinically established ones and others less studied. Regarding CRP, an extensively studied biomarker of systemic inflammation and important predictor of cardiovascular health status, data on whether its level is altered by variations in FV intake are inconclusive. In this respect, our study did not reveal any effect on the levels of CRP by increased FV intake. Likewise, previous RCTs performed in samples of participants with overweight or obesity and increased cardiovascular risk have shown no significant alterations in CRP levels by increased FV intake [15,16,33,34]. In fact, it has been demonstrated that circulating CRP levels are strongly related to indicators of adiposity in older adults [35], even after controlling for variation in physical fitness level [20]. Thus, it can be hypothesized that an intervention-induced change in adiposity level would contribute to alterations in circulating CRP levels. Therefore, the lack of FV-induced changes in central (BMI) and abdominal (waist circumference) indicators of adiposity level may partly explain the lack of beneficial impact on this clinically relevant biomarker of systemic inflammation. Interestingly, data from previous studies have revealed significant effects on the level of the inflammatory biomarker serum amyloid A [16] and improvements in cardiovascular function in response to increased FV [36,37], albeit no effects on CRP were evidenced. Taken together, this indicates that beneficial FV-related effects on the inflammatory environment and cardiovascular health cannot be captured by the sole detection of CRP. Therefore, analysis of a wider selection of inflammatory biomarkers is likely necessary to capture the influence of increased FV intake. Indeed, while no effect on CRP, IL-6, TNF-α, MIP-1α and β, and IL-18 were evidenced, significant reductions in levels of TRAIL, TRANCE, and CX3CL1/fractalkine occurred in participants from the FV group. These factors have been shown to be involved in the regulation of inflammatory pathways implicated in the pathogenesis of various disease conditions. For example, both TRANCE and CX3CL1/fractalkine have been recognized as inflammatory mediators involved in the progression of cardiovascular disease through associations with increased expression of inflammatory factors (Intercellular adhesion molecule-1; ICAM-1, vascular cell adhesion molecule-1; VCAM-1) and roles in platelet activation and monocyte recruitment of the vascular wall [21,22]. Furthermore, serum levels of TRAIL have been shown to be positively correlated with the pro-inflammatory marker TNF-α and inversely associated to an index of lung function in patients with chronic obstructive lung disease, highlighting the implication of TRAIL in disease-associated inflammatory processes [23].

To the best of our knowledge, this is the first study to report on the impact of increased FV intake on these novel biomarkers. Although the exact mechanisms by which fruits and vegetables impact on these biomarkers remain to be determined, it can be hypothesized that the anti-inflammatory properties of FV-related constituents such as potassium, folate, vitamins, fiber, and other phenolic compounds may partly explain the observed effects [38]. Taken together, further research is warranted to clarify the importance of fruit and vegetables for the regulation of the inflammatory environment and its clinical implications for healthy aging.

Our findings are strengthened by the experimental design, where participants were randomly assigned to intervention and control groups. Another important strength is the objective assessment of habitual physical activity level, which enabled depiction of the true effects of dietary changes from those potentially exerted by variations in physical activity. Although all participants in the experimental group did not reach the recommended five FV servings/day, the health education approach successfully generated a two-fold difference in FV intake between intervention groups over a 16-week period, enabling the exploration of FV-related effects on the inflammatory environment. While the dietary counseling and support model promoted dietary changes in our sample of older men and women, its applicability in other populations with various sociodemographic and economic backgrounds needs to be further evaluated. In the present study, we observed changes in macronutrient intake favoring an increased fiber intake similar to previous reports. In this respect, a limitation to this study is that the increased FV intake was not further confirmed using proxy blood markers. Finally, the assessment of dietary changes in our study was based on the use of a validated FFQ [39]. While all dietary assessment tools are prone to misreporting, previous research has shown that FFQs are able to capture changes in dietary intake during intervention studies with an accuracy similar to weighted food record or 24-h recall [40,41].

## 5. Conclusions

The present study adds to current knowledge on the health effects of fruit and vegetables and highlights the occurrence of changes at the level of some inflammatory biomarkers related to increased FV consumption in a population of older men and women. However, the overall clinical implications of FV-induced alterations in these inflammatory biomarkers merit further examination, especially in older populations, where data on links between healthy diet and systemic inflammation remain scarce. 

## Figures and Tables

**Table 1:** Subject characteristics in the fruit and vegetable (FV) and control (CON) groups.

	FV		CON	
	Men	Women	Men	Women
	(*n* = 16)	(*n* = 17)	(*n* = 14)	(*n* = 19)
Weight (kg)	91.7 ± 11.7	69.3 ± 7.2	87.8 ± 7.8	71.9 ± 5.6
Height (cm)	181 ± 7	164 ± 6.6	179 ± 7.5	166 ± 4.5
BMI (kg/m^2^)	27.9 ± 3.1	25.9 ± 2.8	27.5 ± 2.1	26.2 ± 1.9
Waist circumference (cm)	103 ± 9.4	86 ± 4.7	102 ± 5.7	87 ± 5.5

**Table 2:** Average number of servings per day of fruit and vegetables in both groups before and during the intervention.

	Before Intervention		During Intervention	
	FV	CON	FV	CON
Fruit and vegetable (servings/day)	2.2 ± 1.3	2.2 ± 1.4	4.2 ± 1.8 *	2.6 ± 1.6

FV; fruit and vegetable group, CON; control group. * significant difference within group (*p* < 0.05).

**Table 3:** Total energy and macronutrient intakes before (PRE) and after (POST) the 16-week period.

	PRE		POST	
	FV	CON	FV	CON
Total energy (kcal)	1699 ± 427	1816 ± 544	1861 ± 474 *	1797 ± 523
Carbohydrates (g)	166 ± 46	180 ± 59	197 ± 63 *	174 ± 52
Fiber (g)	23 ± 10	26 ± 11	29 ± 12 *	26 ± 10
Protein (g)	68 ± 19	71 ± 23	73 ± 20	71 ± 22
Fat (g)	77 ± 24	82 ± 28	79 ± 25	81 ± 29

FV; fruit and vegetable group, CON; control group. * significant difference within group (*p* < 0.05).

## Data Availability

Data supporting the reported results are available upon reasonable request and in accordance with the ethical principles.

## Supplementary Materials

The following are available online at https://www.mdpi.com/article/10.3390/nu13113753/s1, Figure S1: Effect of intervention on IL-6 (a), IL-18 (b), MIP-1α (c), MIP-1β (d), and TNF-α (e). FV; fruit and vegetable group, CON; control group. No significant time × group interaction was denoted.
