# Supplementary material for: Randomized Controlled Trial for Promotion of Healthy Eating in Older Adults by Increasing Consumption of Plant-Based Foods: Effect on Inflammatory Biomarkers
Source: Nutrients. 2021 Oct 24;13(11):3753. doi: 10.3390/nu13113753 (PMC8623008; doi:10.3390/nu13113753)
Supplement: Supplementary file 1 [file nutrients-13-03753-s001.zip › nutrients-1417947-supplementary.pdf]

nutrients

Supplementary Materials

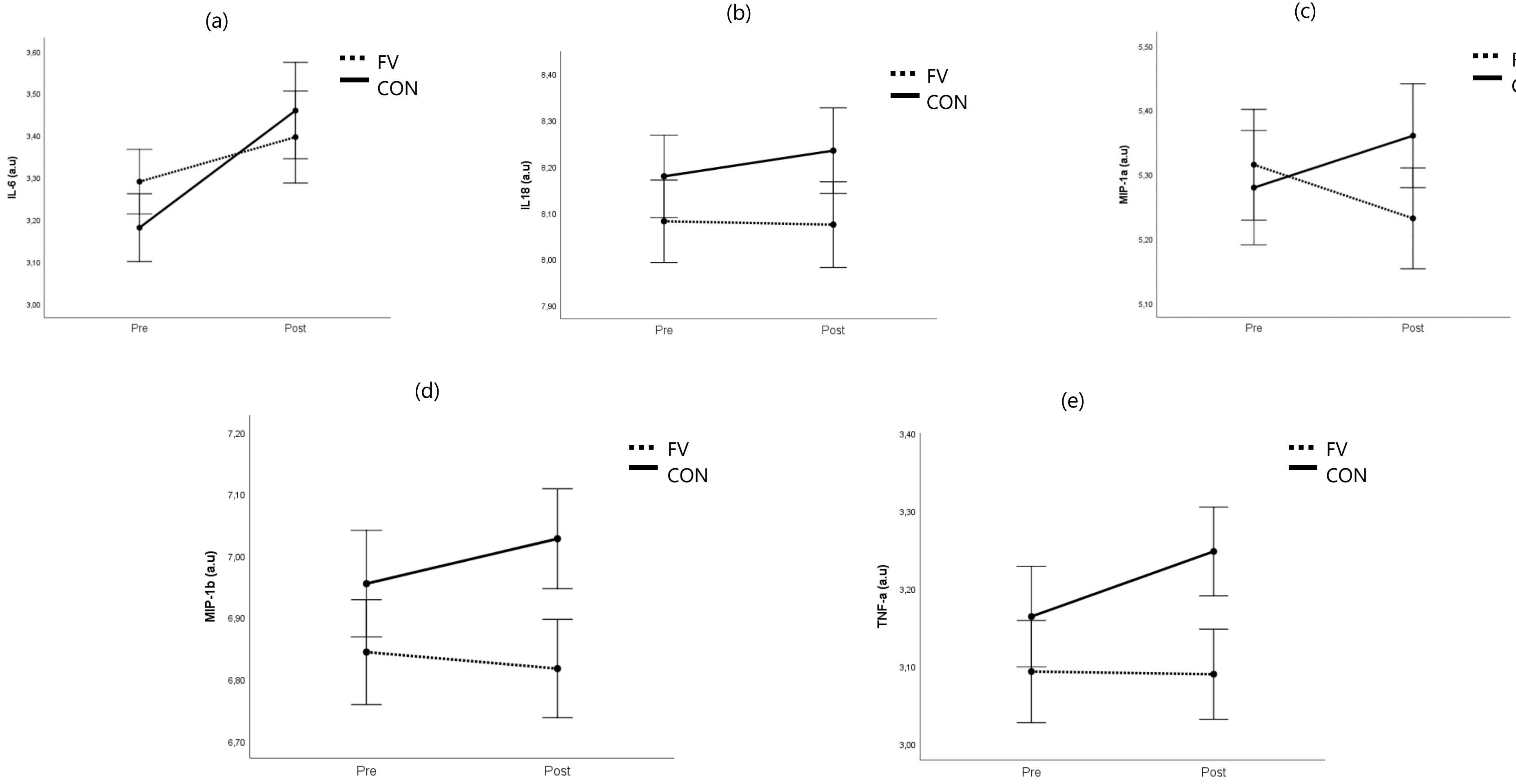

Supplementary Figure S1. Effect of intervention on IL-6 (a) , IL-18 (b), MIP-1α (c), MIP-1β (d) and TNF-α (e). FV; fruit and vegetable group, CON; control group. a.u; arbitrary units. No significant time x group interaction was denoted.
